# Latent Space Search-Based Adaptive Template Generation for Enhanced Object Detection in Bin-Picking Applications

**DOI:** 10.3390/s24186050

**Published:** 2024-09-19

**Authors:** Songtao Liu, Yaonan Zhu, Tadayoshi Aoyama, Masayuki Nakaya, Yasuhisa Hasegawa

**Affiliations:** 1 Department of Micro-Nano Mechanical Science and Engineering, Nagoya University, Nagoya 464-8601, Japan; aoyama@mein.nagoya-u.ac.jp (T.A.); hasegawa@mein.nagoya-u.ac.jp (Y.H.); 2 The School of Engineering, The University of Tokyo, 7-3-1 Hongo, Bunkyo-ku, Tokyo 113-8656, Japan; yaonan.zhu@weblab.t.u-tokyo.ac.jp; 3 Robot Division, System Department, NACHI-FUJIKOSHI CORP., Toyama 930-8511, Japan; masayuki.nakaya.ho@nachi.com

**Keywords:** template matching, template searching, template generation, bin picking

## Abstract

Template matching is a common approach in bin-picking tasks. However, it often struggles in complex environments, such as those with different object poses, various background appearances, and varying lighting conditions, due to the limited feature representation of a single template. Additionally, during the bin-picking process, the template needs to be frequently updated to maintain detection performance, and finding an adaptive template from a vast dataset poses another challenge. To address these challenges, we propose a novel template searching method in a latent space trained by a Variational Auto-Encoder (VAE), which generates an adaptive template dynamically based on the current environment. The proposed method was evaluated experimentally under various conditions, and in all scenarios, it successfully completed the tasks, demonstrating its effectiveness and robustness for bin-picking applications. Furthermore, we integrated our proposed method with YOLO, and the experimental results indicate that our method effectively improves YOLO’s detection performance.

## 1. Introduction

Template matching is a common solution for the bin-picking task [1], and it is also a widely adopted technique in many fields such as industrial manufacturing [2,3] and robot manipulation [4,5], utilized for identifying and locating similar patterns within images. This method involves comparing small patches or regions of an image to a reference template, aiming to detect corresponding features. Typically, measures such as normalized cross-correlation or correlation coefficients are employed to assess the similarity.

Challenges arise when objects are within cluttered environments, presenting various poses [6], background appearances [7], and illumination conditions [8]. A single template may not capture the full variability of an object’s appearance under different circumstances, limiting its performance in diverse settings. Image processing techniques can enhance the matching performance by providing a single template with various feature representations. For example, rotating templates to detect objects in different orientations can be effective. However, in scenarios with complex backgrounds or significant variations in poses, traditional template matching approaches may struggle to maintain high performance. These situations are challenging to characterize and can hinder the effectiveness of image processing approaches, making it difficult to achieve accurate detection in such challenging environmental conditions.

One approach to tackle this issue is by creating a diverse set of templates and employing them for template matching [9,10]. Nevertheless, the variability is boundless, making it difficult to gather infinite possible templates, and searching through such a vast dataset would be challenging [11]. Although some linear template searching methods, such as [12], have been introduced to improve computation efficiency, linear spaces like pose space may not capture all the features of the templates. The object’s appearance can be affected by the environment, such as background appearance and reflection, leading to mismatches. To improve the conventional linear template searching strategy for effectively generating an environmentally adaptive template, we outline the following requirements:

- Complex Feature Characterization for Templates: The complex features of a template, such as background appearance and surface reflection of the object, are challenging to characterize using linear features. Therefore, a method for complex feature characterization is needed to induce a compact feature space for effective template searching.
- Effective Searching Method in Feature Space: The feature space formed by complex template features is often nonlinear. An effective searching method is needed to navigate and localize an environmentally adaptive template’s features within this nonlinear space.

To address these requirements, this study employed the following approaches:

- Template Feature Characterization by VAE: A Variational Auto-encoder (VAE) [13] has the capability to effectively capture variations in training data and represent them in a latent space [14]. This latent space can be treated as a Gaussian distribution, enabling the VAE to characterize the features of templates into a low-dimensional latent space that encapsulates all variations of the templates, facilitating the subsequent searching step. This latent space can be considered as the templates’ feature space.
- Adaptive Template Searching by CEM: The Cross-Entropy Method (CEM) [15,16] adaptively refines the sampling distribution towards areas with higher rewards. By focusing on the best-performing samples in each iteration, the CEM effectively narrows down the search space, leading to more efficient policy optimization. Therefore, it is adept at navigating an adaptive latent vector in a nonlinear space [17,18], making it suitable for generating adaptive templates that can dynamically adjust to varied environmental conditions.

**Figure 1:**
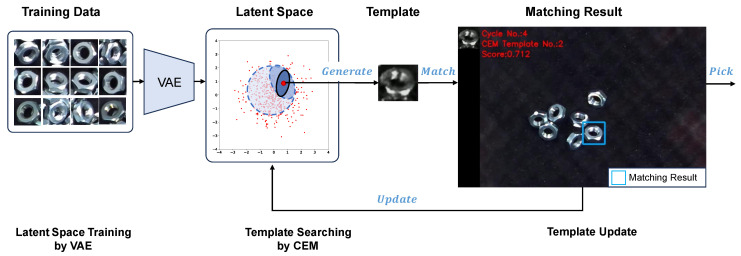
The framework of a variational template generation control system. Firstly, utilizing a VAE to train a low-dimensional latent space to represent the templates’ feature map of the training data. Then, employing a CEM to identify an adaptive template within the latent space. Lastly, updating the template based on its matching performance feedback. In the bin-picking task, the image changes after each object is picked. If the current template’s matching performance falls below a predefined threshold, the template will be updated based on the current camera’s frame.

The integration of a VAE and CEM facilitated the development of a variational template generation control system (Figure 1). This method allows for the generation of adaptive templates that dynamically adjust to the current environmental conditions, thereby enhancing the performance of object detection systems in varied scenarios.

## 2. Literature Review

Template matching algorithms heavily depend on the similarity measure used to align the template with a candidate window in the target image. Various similarity measures have been applied for this purpose, including the widely used Sum of Squared Differences (SSD), Sum of Absolute Differences (SAD), and Normalized Cross-Correlation (NCC). Despite the computational efficiency of these conventional methods [19], those methods may struggle to effectively detect objects in scenarios involving significant deformations in pose, background, illumination, etc., within the object window.

Considerable efforts have been directed towards enhancing the performance of the similarity measure to address these challenges. Researchers have explored various strategies to improve the similarity measurement. Korman et al. developed a matrix called “Fast-Match”, a fast algorithm for approximate template matching under 2D affine transformations to counter complex geometric deformations in the target image [6]. Oron et al. proposed Best-Buddies Similarity (BBS) that makes it effective against complex geometric deformations and background clutter [7]. Hel-Or et al. designed a “Matching by Tone Mapping (MTM)” measure which is insensitive to the illumination [8].

Another approach to improving matching performance involves utilizing “features”. While traditional template matching methods directly compare the pixels of the template and the target image, an alternative approach is to extract features, such as feature points [20] and color feature [21], from the images and perform matching based on these extracted features. This method can enhance performance by focusing on the distinctive characteristics of the images rather than directly comparing the pixel values.

Matching with image features allows for more robust matching against variations such as changes in lighting, scale, rotation, and perspective. Techniques such as feature extraction using methods like SIFT (Scale-Invariant Feature Transform) [20] and SURF (Speeded Up Robust Features) [22] have shown significant improvements in matching accuracy and robustness in various computer vision applications.

While these methods have indeed improved the performance of template matching, they may still face challenges when confronted with large deformations. One reason for this limitation is that a single template (image, point, histogram, etc.) only captures a specific feature of the object. However, the environment is typically dynamic, leading to variations in the features that the set template finds difficult to handle. Consequently, the template needs to be continually updated to adapt to changes in the environment. This reliance on manual template adjustments poses a significant challenge, requiring human intervention to ensure accurate matching.

Combining multiple templates can be a viable approach to address the limitations of using a single template, especially for objects with geometric variations. Although matching with multiple templates has been shown to improve matching performance [9,10], searching for an adaptive template from the vast dataset effectively poses challenges. Conventional template searching approaches usually generalize templates as a linear space such as 3D pose space and search in this linear space. For instance, Ye et al. (2016) [12] prepared templates with different viewpoints of the object and proposed a hierarchical template searching strategy that searches for an adaptive template at different feature density levels of the 3D pose space. However, a linear space may not induce all template features, especially in a cluttered environment, in which an object’s appearance can be affected.

Different from searching in a linear feature space, our proposed method employs a latent space trained by a VAE for template searching. The training data contained various features, and the VAE extracted these features into a low-dimensional latent space. This latent space serves as a more effective representation for capturing the diverse range of object variations encountered in real-world scenarios. Furthermore, based on the unique characteristics of the latent space, which is represented by a Gaussian distribution, we integrated the CEM to localize an adaptive template representation within the latent space.

## 3. Materials and Methods

### 3.1. Environmental Setup

**Figure 2:**
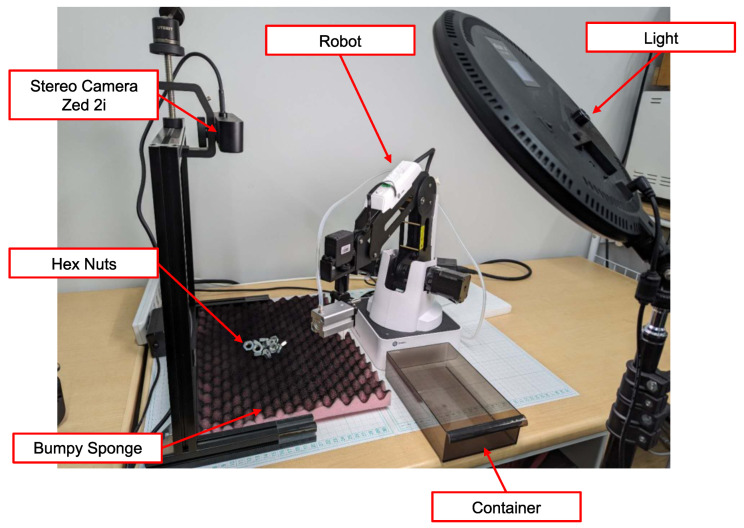
Environmental setup. Hex nuts were utilized as the research subject of the study. To demonstrate various poses, a bumpy sponge served as the foundation. Image capture and depth detection were facilitated by a stereo camera system. The lux level of the workspace was set as 1200 lux.

In Figure 2, the overall environmental setup is depicted. Hex nuts were selected as the focus of our study due to their susceptibility to geometry deformation caused by pose variations and their sensitivity to changes in illumination. Moreover, their hollow interior leads to varying background appearances, posing additional challenges for object detection. To present a diverse range of poses, we utilized a bumpy sponge as the foundation and applied black paint to minimize noise from the foundation’s shape. The light intensity was set as 1200 lux, according to the lux standard of the Japanese Industrial Standards (JIS).

A stereo camera (ZED 2i, Stereolabs, San Francisco, CA, USA) was employed for image capture and depth detection with 1080P resolution and 15 fps. The left lens (Focal Length 2.1 mm) was utilized for image capture, and its neural depth mode was used to obtain the depth of the detected object. The camera’s depth range corresponds to the minimum and maximum distances, which are 0.3 m to 20 m, and the distance between the camera and workspace was set as 0.33 m.

**Figure 3:**
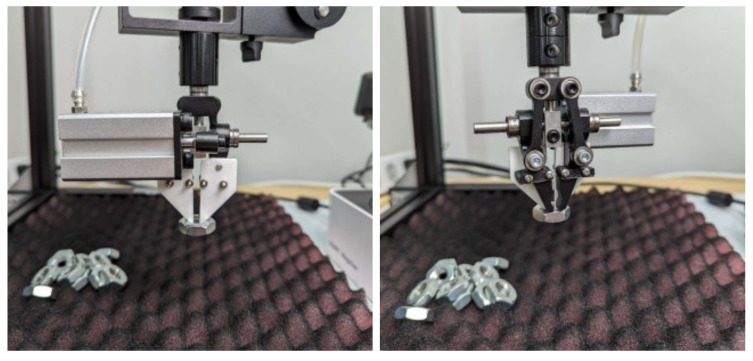
Grippers designed for nut grasping.

A robot (Magician, Dobot, Shenzhen, China) was equipped with grippers specifically designed for nut grasping, as illustrated in Figure 3. The robot’s workspace was defined by its movable range and was cropped from the entire camera frame to focus on the relevant area, with the top left corner of the cropped image serving as the origin of the workspace.

To ensure accurate positioning, the system was calibrated based on this origin. The XY coordinates of the objects were determined using template matching, and these coordinates were translated to the robot’s coordinate system. The Z position was measured by obtaining the depth of the center of the detected object window using the camera’s neural depth mode.

### 3.2. Data Collection

The data collection method, inspired by the data engine approach outlined in [23], ideally consists of three progressive steps: assisted-manual, semi-automatic, and fully-automatic. In this study, data were collected by the assisted-manual and semi-automatic methods.

**Figure 4:**
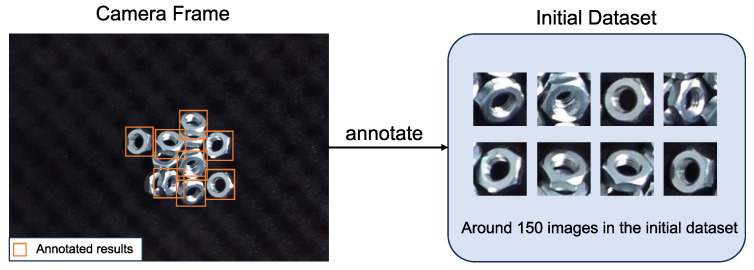
Assisted-manual data collection stage. In this stage, operators are directly involved in annotating objects in the initial dataset. An object that can be grasped by the robot will be annotated. Additionally, when the model fails to detect objects in certain scenes, manual annotation is used to collect data for those instances.

**Figure 5:**
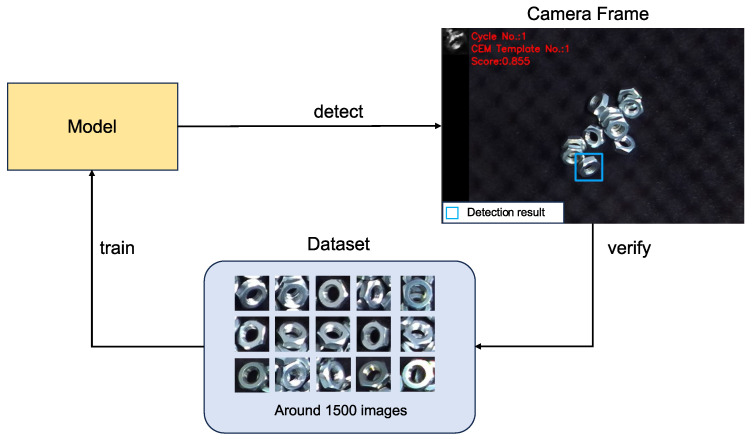
Semi-automatic data collection stage. In this stage, the model is deployed to automatically detect objects. However, due to potential limitations in the model’s performance, human verification is required to confirm the accuracy of the detected objects. Any discrepancies or inaccuracies identified during this process are annotated and corrected to ensure the quality of the training dataset.

- Assisted-Manual: In the initial stage of the training process, a set of initial data was collected to train a basic model. During this step, the operator manually annotated the results from the camera frames. An object that can be grasped by the robot will be annotated. The annotation window’s size is fixed at 64 × 64 pixels, with the center of the window representing the robot’s grasping point. This manual intervention ensures the availability of comprehensive training data, aiding in the improvement in the model’s performance over subsequent iterations (Figure 4).
- Semi-Automatic: Following the development of a basic model, it was deployed for experimentation. Each detected object was picked by the robot and the results were recorded. Given that the model was trained with limited data, it may not consistently produce accurate results or may miss detections entirely. Hence, human annotation was required to verify the recorded results, as well as annotate scenes where the model failed to detect objects. This iterative approach helps refine the model’s capabilities and improve its performance over time (Figure 5).
- Fully-Automatic: Once the model has been trained on a large dataset and achieved high performance, it can proceed to the fully automatic stage. In this phase, the model autonomously detects objects, the robot picks them, and data recording occurs automatically.

Following the approach of the data engine, we collected over 1500 images, each with dimensions of 64 × 64 pixels, for model-training purposes.

### 3.3. Latent Space Learning by VAE

The VAE was trained for latent space learning, where the variations present in the training data, including pose, background, illumination, etc., were compressed into a latent space. This process enables the model to capture the essential characteristics of the data while reducing its dimensionality, facilitating more efficient representation.

**Figure 6:**
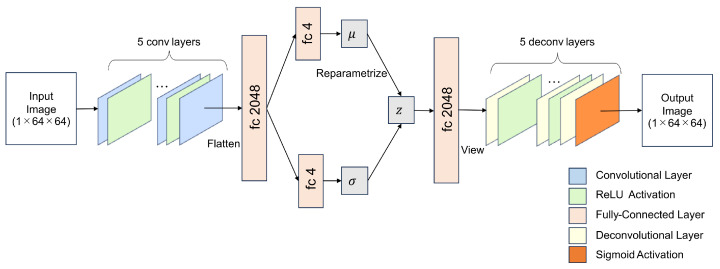
The architecture of a VAE training model. Input: 1×64×64 grayscale image. Encoder: 5 convolutional layers with ReLU activation, and the last convolutional layer is flattened as a 2048-neuron fc layer. Latent vector *z* is 4-dimensional, obtained by 2 fc layers representing mean μ and variance σ using the reparameterization trick. Decoder: Latent vector mapped to a 2048-neuron fc layer, then reshaped to (512, 2, 2) size by using the “view” function of the Pytorch. Followed by 5 deconvolutional layers with ReLU activation. The last deconvolutional layer is activated by Sigmoid to obtain the output image. Output: 1×64×64 grayscale image.

The architecture of the VAE training model is illustrated in Figure 6 and the model was trained by Pytorch. Input images were 1×64×64 grayscale images, as template matching was performed using grayscale images. The encoder part of the VAE comprised five convolutional layers and four ReLU activation layers. The final convolutional layer was flattened into a fully connected (fc) layer with 2048 neurons, followed by 2 additional fc layers with 4 neurons each. The reparameterization trick [13] was then employed for the latent vector *z*, with the dimension of the latent vector set as four, sampled from a Gaussian distribution.

In the decoder part, the latent vector was mapped to a 2048-neuron fc layer followed by 5 deconvolutional layers and 4 ReLU activation layers. The final convolutional layer was activated by Sigmoid to generate the image. During training, the VAE was optimized by minimizing the reconstruction error using mean square error (MSE) and the KL divergence with the Adam optimizer [24]. Consequently, a four-dimensional latent space was established, modeled as a multidimensional Gaussian distribution. The specifications of the model-training computer included an Intel i7-10700 CPU and an NVIDIA GTX 1660S with 6 GB of VRAM.

### 3.4. Template Searching by CEM

The latent space trained by the VAE encapsulates the variations present in the training data, with each latent vector representing a distinct latent feature of the training data. However, each dimension of the latent space is correlated, unlike a typical Euclidean space where each dimension is independent. Therefore, identifying a specific template’s latent vector to correspond with the environment is challenging.

Although in recent years, disentangled VAEs such as β-VAE [25] and FactorVAE [26] have been proposed to ensure that each dimension in the latent space corresponds to a distinct and interpretable factor of variation in the data, some limitations exist for the image generation. The main idea is to increase the coefficient of the KL term in the VAE’s training loss to achieve higher disentanglement, but this results in a higher reconstruction error compared to a standard VAE [26], which may affect the matching results due to the inaccurate template generation. Furthermore, it is challenging for the training data to cover all possible factor combinations. For inputs with untrained factor combinations, disentangled VAEs may struggle to accurately generate images [26].

The CEM is a Monte Carlo method for importance sampling and optimization, frequently applied in nonlinear problems such as robot motion planning [17,27]. In this study, the objective of the CEM is to find a latent vector with an optimal factor combination, allowing the generated template to accurately reflect the ground truth of the input.

**Figure 7:**
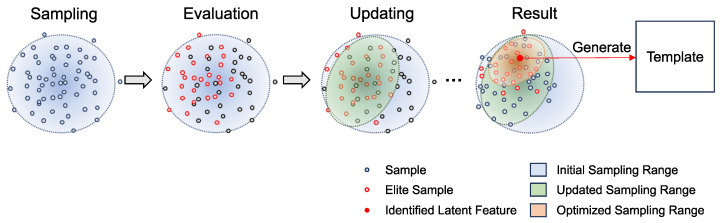
The template searching process by CEM. The search process by CEM involves sampling, evaluation, and updating. Sampling: samples are drawn from a Gaussian distribution. Evaluation: each sample point is evaluated, and elite samples are selected based on their evaluation results. Updating: the sampling range is updated based on the elite samples. This process is repeated until the sampling range becomes smaller than a predefined threshold. The mean of the optimized sampling range is then used as the identified latent feature to generate the template for matching.

Figure 7 illustrates the template searching process by the CEM, where each optimization cycle involves three steps: sampling, evaluation, and updating.

1. Sampling: The trained latent space is modeled as a Gaussian distribution, and the samples $P_{k=1\ldots K}$ of the initial cycle are drawn from this Gaussian. Here, *K* represents the number of the samples.
  (1) $$P_{k=1\ldots K}∼N\left(\mathrm{mu},\;\mathrm{var}\right)$$
  Here, mu is the mean and var is the covariance matrix of the Gaussian, respectively. For the initial step of the CEM, the mu is the zero-matrix and var is the identity matrix.
2. Evaluation: Each sampled point is evaluated, and elite samples are selected to form a new Gaussian distribution for optimizing the sampling range. Each sampled point, which can be treated as a latent vector, is used to generate template candidates $T_{k=1\ldots K}$ by the VAE’s decoder *f*,
  (2) $$T_{k}=f\left(P_{k}\right)$$
  and the Normalized Cross-Correlation (NCC) is employed as the evaluation metric due to its computational efficiency.
  (3) $${\mathrm{score}}_{k}=ncc\left(T_{k},\;I\right)$$
  Here, ${\mathrm{score}}_{k}$ represents the evaluation result which is calculated by the maximum cross-correlation between the template candidate and the target image. Subsequently, sampled points are sorted based on the evaluation result.
  (4) $$sort:P_{k=1\ldots K}\leftarrow P_{k=1\ldots K}\;w.r.t\;{\mathrm{score}}_{k=1\ldots K}$$
3. Updating: Based on the sorted results, $K_{e}$ number of elite samples are selected to form the new sampling range, and form a new Gaussian’s mean mu′ and covariance matrix var′. After the updating, new samples will be sampled from this new Gaussian distribution.
  (5) $${\mathrm{mu}}^{\prime }=\sum_{k=1}^{K_{e}}\frac{1}{K_{e}}P_{k}$$
  (6) $${\mathrm{var}}^{\prime }=\sum_{k=1}^{K_{e}}\frac{1}{K_{e}}\left(P_{k}-\mathrm{mu}\right){\left(P_{k}-\mathrm{mu}\right)}^{T}$$

**Figure 8:**
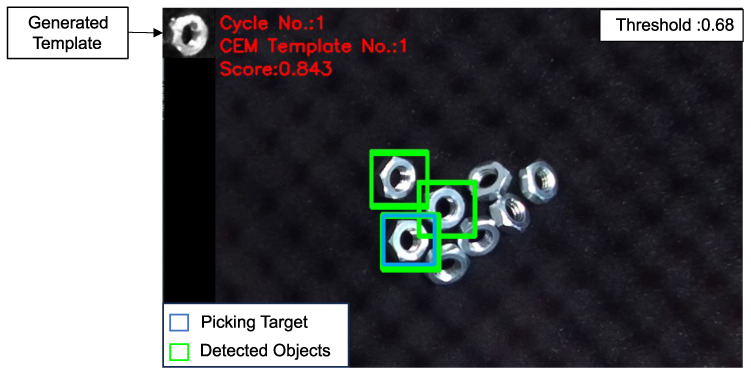
Generated template and its matching result. The green windows indicate the detected objects with a matching score above the threshold of 0.68. Meanwhile, the blue window highlights the location with the highest matching score, serving as the picking target for the robot.

**Figure 9:**
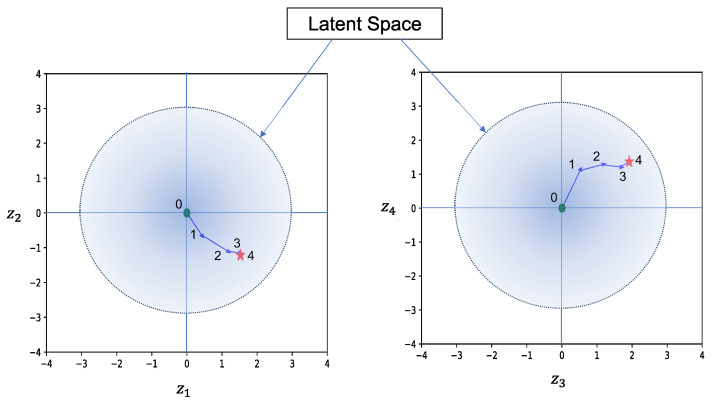
Searching process in latent space. In the figure, $z_{k}\left(k=1,\;2,\;3,\;4\right)$ represents each dimension of the latent space, the green point shows the start, and the red star shows the finish.

**Figure 10:**
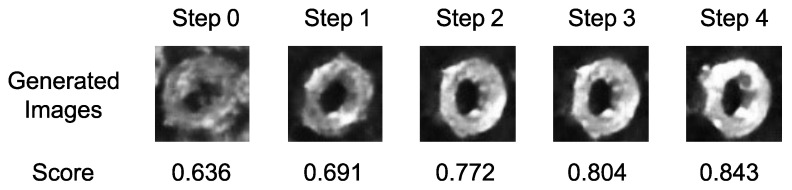
The series of generated templates and their corresponding matching scores at each searching step. Initially, the start latent vector produces an image of the nut with unclear features. As the CEM progresses, the generated image evolves, and its matching score increases.

Here is an example illustrating how the CEM generates the template. In Figure 8, the generated template and its corresponding matching result are displayed. The process begins with the CEM navigating the latent vector from the origin point of the latent space to an adaptive latent feature, as shown in Figure 9. For each representation of the sampling range, Figure 10 illustrates the series of images generated by the mean of the sampling Gaussian distribution of each step, demonstrating the effectiveness of the adaptive template generation process.

In this study, the threshold was set at 0.68, considering both the quality of image generation by the VAE and the need to distinguish objects from the environment.

### 3.5. Template Update

**Figure 11:**
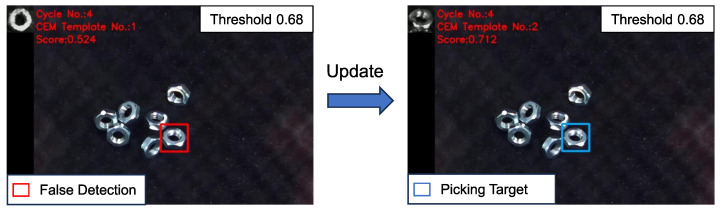
Example of template updating during robot bin-picking. The previous template was generated as planned, but the objects in the image were all tilted, resulting in false-negative detection (the matching score was 0.524, smaller than the threshold of 0.68). After the template was updated using the CEM method, the matching performance improved significantly, with a matching score of 0.712, indicating successful object detection.

As the robot picks the detected objects, the image changes and the performance of the previous template may fall below the threshold. In such cases, the template needs to be updated. Figure 11 gives an example of template updating, illustrating how the updated template improves the matching performance compared to the previous template.

In this study, 150 samples were generated for each step of the CEM process, with the top 30% samples selected as elite samples. The CEM search process continued when the covariance matrix determinant of the updated Gaussian distribution was reduced to less than 20% of the initial. Template candidates’ images were generated simultaneously using CUDA, while each candidate was matched with the camera frame.

## 4. Experiment Validation

### 4.1. Validation for Template Searching Efficiency

To evaluate the efficiency of proposed method, we used a group of nuts under various conditions, including different poses, backgrounds, and lighting conditions. The experimental setup remained consistent with the data collection environment depicted in Figure 2, and the same computer used for model training was employed.

In the experiments, once an object is detected, the robot performs pick-and-place actions and proceeds to the next detection task. If the matching score falls below 0.680, indicating a potential mismatch, the template is updated. If the matching score remains below the threshold for five consecutive detections, the system infers that an adaptive template cannot be generated to detect any objects and halts the operation. A total of 25 nuts were used for each trial. The task performance was evaluated with the following metrics:

1. Success Rate: The percentage of successful grasping attempts. Picking failures caused by hardware issues, such as the camera’s depth detection errors or accidental dropping from the gripper, were not considered as failed grasping attempts.
2. Percent Cleared: The fraction of objects that were moved to the container.
3. Picks per Hour (PPH): The estimated number of bin picks per hour of runtime, which is computed by multiplying the number of successful grasping attempts per hour.

In contrast with linear template searching at different levels of the dataset, we randomly selected 150, 300, 600, and 1500 templates from the total dataset which was used for latent space training. The template update times for each template searching strategy are shown in Table 1.

#### 4.1.1. Different Object’s Poses

**Figure 12:**
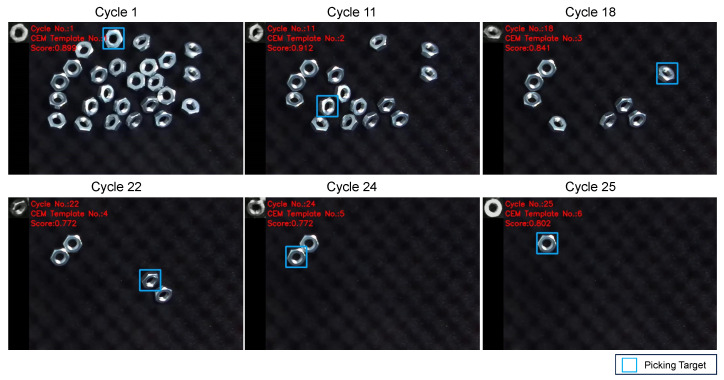
Snapshots of the experiment with different object’s poses. In the experiment, all the objects were successfully detected and picked up.

**Figure 13:**
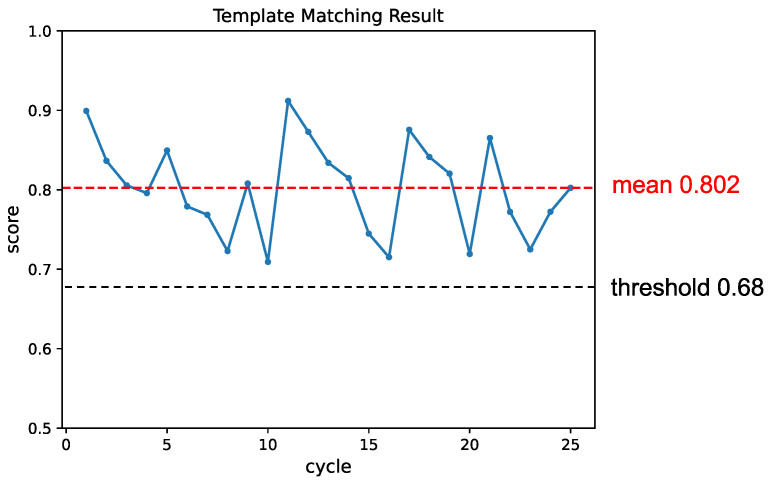
The change in matching score throughout the experiment. Each instance where an object was detected and picked up was considered one cycle. The template was updated whenever the matching score fell below the threshold, leading to a subsequent recovery in the matching result.

Firstly, we validated the detection performance of objects in various poses. Figure 12 illustrates snapshots from the experiment, demonstrating the detection of objects in different poses. Figure 13 shows the change in matching scores during the experiment. The matching score of the generated template decreases as the object is picked up. When the performance falls below the threshold, the template is updated, leading to a recovery in the matching score.

Table 2 presents the results of the experiment using the proposed method, and Table 3 illustrates the comparison with different numbers of templates. The templates were randomly selected from the total dataset, and each searching strategy was tested under the same conditions as the proposed method.

In this condition, almost all strategies successfully cleared the task. The searching strategy using 150 templates failed to detect all objects in some situations due to the limited number of templates. In different levels of template data, as the number of templates increased, the PPH decreased because the searching process during template updates took more time. From the experimental results, the proposed method achieved a moderately high level compared to others.

#### 4.1.2. Objects with Varying Background Appearances

**Figure 14:**
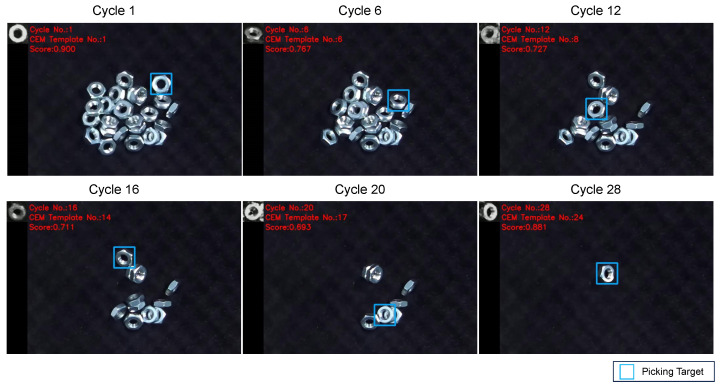
Snapshots of the experiment with varying background appearances. The significant variations in each window candidate necessitated frequent updates to the template.

**Figure 15:**
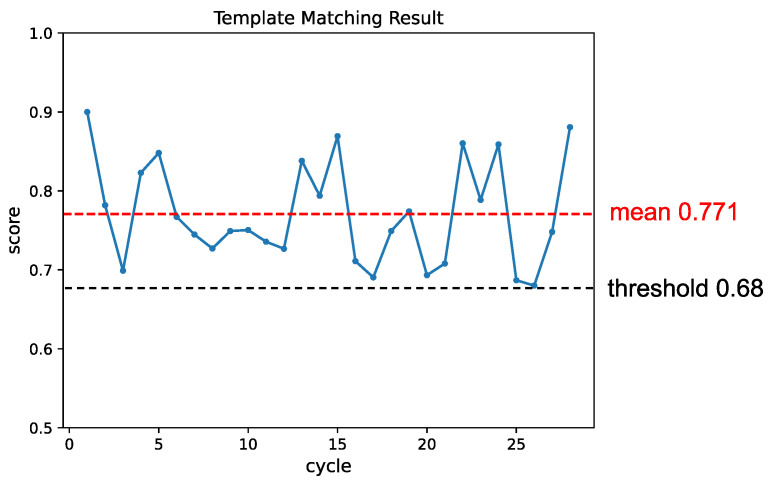
The change in matching score throughout the experiment in varying background appearance conditions. Similarly to the pose condition experiment, the template was updated whenever the matching score dropped below the threshold, resulting in subsequent improvements in the matching results.

To create varying background appearances, we intentionally overlapped objects. Due to the significant variations in each object window, the template needed frequent updates to maintain matching performance as shown in Figure 14. The matching score’s change was shown in Figure 15, and each cycle’s matching score remained above the threshold.

**Figure 16:**
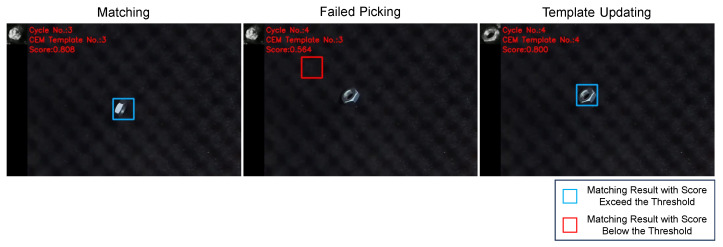
Snapshots of object detection and picking process in a vertical pose. Matching: As the vertical pose was untrained, the proposed method generated templates with similar features but did not accurately resemble the shape of a nut. Despite this discrepancy, the template detected the object. Failed Picking: Due to the limitation of the robot’s gripper, the robot knocked down the nut. Template Updating: Since the object’s pose changed and the matching score fell below the threshold, the template was updated accordingly, and the robot proceeded to pick up the nut.

During the experiment, two typical unexpected scenarios led to failed grasping attempts. Figure 16 illustrates an example of the detection and picking process for an object in a vertical pose. Despite this unforeseen circumstance, where the vertical pose was not included in the trained dataset due to limitations in the robot gripper, our proposed method detected objects with vertical poses. However, in this untrained situation, the generated template did not accurately resemble a nut, resulting in a failed grasping attempt.

**Figure 17:**
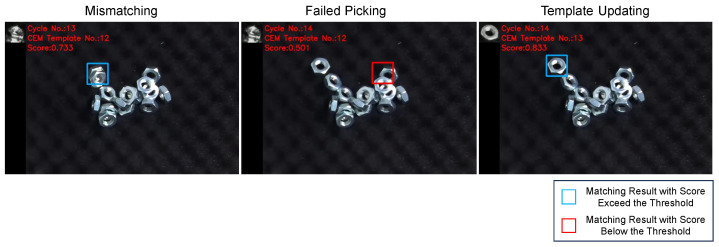
A failed grasping attempt case by low-precision matching result. Mismatching: The NCC similarity measure does not effectively capture the spatial relationships between objects and led to failure. Failed Picking: The robot failed to grasp the nut accurately, causing it to move. Template Updating: The template was updated, improving the matching result.

In another instance of a failed grasp attempt caused by the limitation of the NCC similarity measure, depicted in Figure 17, the template achieved a matching score above the threshold. However, an overlapped object was detected, resulting in a failed grasping attempt. This failure highlights that the NCC similarity measure does not effectively capture the spatial relationships between objects.

Table 4 summarizes the experiment results, and Table 5 compares the results using different strategies. As the number of templates increased, an increase in percentage cleared and a decrease in PPH were observed simultaneously. The increase in the number of templates allowed more features to be detected, enhancing the percentage cleared. On the other hand, an excessive number of templates caused the PPH to fall due to the longer searching process during template updates. In this complex environment, our proposed method maintained the highest PPH compared with others.

#### 4.1.3. Evaluation with Additional Lighting Source

**Figure 18:**
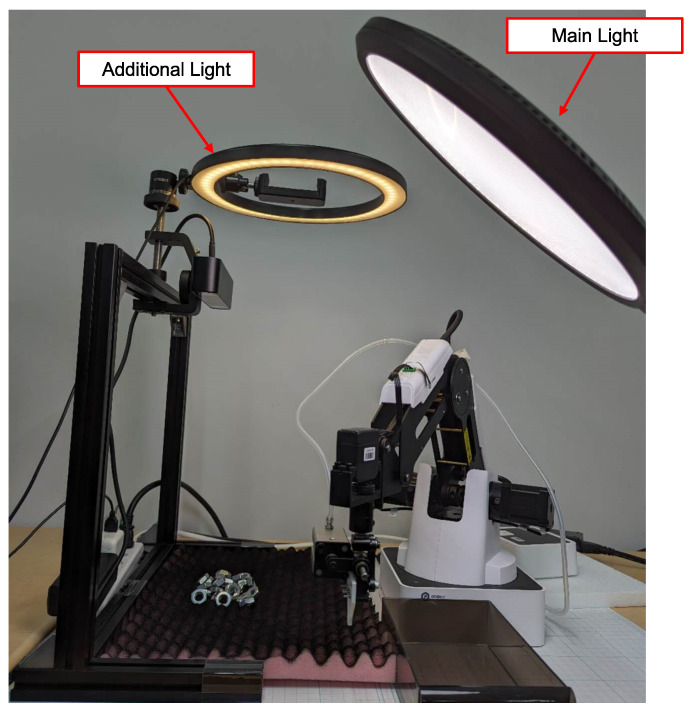
Experimental setup with additional lighting source. In addition to the existing lighting conditions, we introduced an additional light source. Furthermore, we altered the color of this additional light to provide a variation from the training environment. The lux level was increased to 1500.

We introduced an additional lighting source (Figure 18) and modified its color to introduce a deviation from the training environment. The experimental procedure was identical to the experiment with the condition described in Section 4.2.

**Figure 19:**
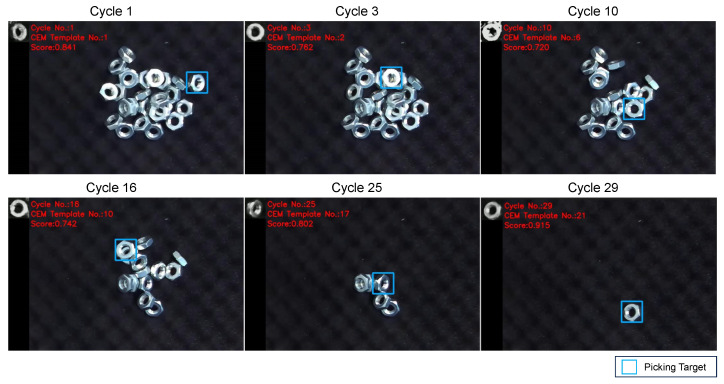
Snapshots of the experiment with an additional lighting source. Despite the change in lighting condition, the object was successfully detected and grasped.

**Figure 20:**
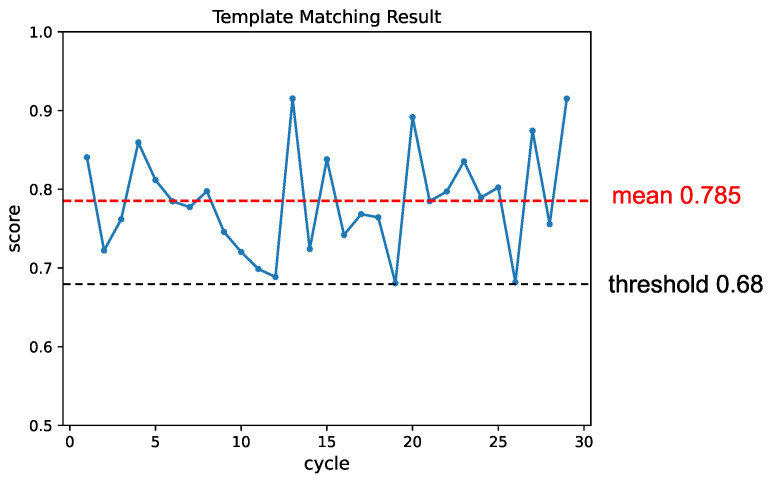
The change in matching score throughout the experiment of lighting condition. Similar to the conditions of pose and background, the template was updated whenever the matching score fell below the threshold, resulting in a subsequent recovery in the matching result.

Experiment results and the changing of the matching score are shown in Figure 19 and Figure 20. Table 6 summarized the results of the experiments in the additional lighting source condition and Table 7 illustrates the experiment results by different strategies.

Due to the influence of the lighting condition, the success ratio and percentage cleared decreased compared to the condition in Section 4.2, especially with a smaller number of templates. We also observed that an excessive number of templates caused the PPH to fall. Despite the changes in lighting conditions, our proposed method still demonstrated higher efficiency.

### 4.2. YOLO Detection Enhancement through Latent Space Template Search

YOLO (You Only Look Once) [28] is a powerful and mainstream object detection model that can be customized by users to detect various objects. However, due to the limited size of the customized training data, the model may not learn all possible input factors and combinations, leading to the detection results of target object in certain states being ambiguous and negatively impacting detection performance [29]. Furthermore, since YOLO detects objects based on deep features trained by neural network, it is difficult to know which factor the model has learned, which can lead to mismatches for objects with similar deep features.

To improve performance with limited customized training data, we integrated YOLO with our proposed latent space template searching method, which generates adaptive templates to refine YOLO’s results through template matching which measures the difference in pixel features. In the experiment, over 200 camera frames with approximately 1500 labeled nut images were used for training both YOLO and the latent space.

The YOLOv8n model was used in the experiment and new latent space was trained in this section. Since the labeled data varied in size, they were resized to 64 × 64 pixels. The same VAE structure was used for latent space training in this research.

#### 4.2.1. Validation with the Coin

**Figure 21:**
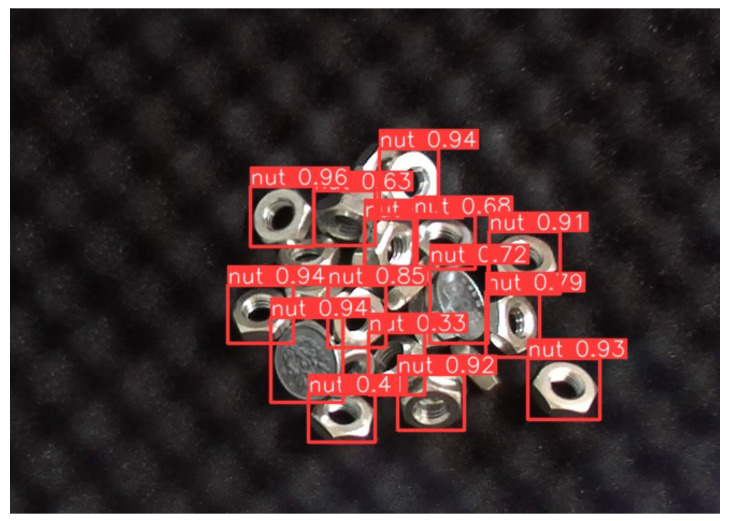
YOLO’s detection results with 100-yen coin. Coins were mistakenly identified as nuts, with confidence scores exceeding 0.90, even higher than those for actual nuts. Additionally, for some nuts, the model failed to achieve a confidence score above the 0.5 threshold.

**Figure 22:**
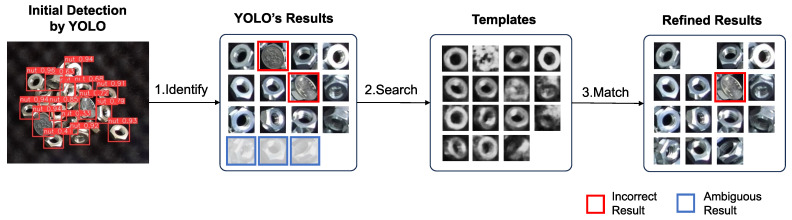
YOLO’s detection results refining process by templates generated via latent space searching. The process involves three steps: (1) Initial detection by YOLO: YOLO identifies potential detection target windows. Incorrect results and ambiguous results which the confidence score under the threshold were observed. (2) Template search based on YOLO’s results: for each potential target, a corresponding template is searched in the trained latent space. (3) Matching with templates: YOLO’s results were refined by template matching. Matches with scores below the threshold are filtered out. Although one different object could not be filtered out by the threshold, its YOLO detection result with a confidence score of 0.717 was refined to a template matching score of 0.567, bringing it closer to the ground truth.

Since a coin has a similar appearance to a nut, we used coins as interfering objects in the experiment. Figure 21 shows the YOLO’s detection result and Figure 22 illustrates the hybrid system for YOLO’s result refining. Two 100-yen coins were used for evaluation, in which the coin was outside the training domain. The threshold of both YOLO and template matching was set at 0.5, based on the PASCAL Visual Object Classes (VOC) metric [30].

YOLO was used for initial detection to identify potential target object candidates. However, YOLO mistakenly identified coins as the target nut with a high confidence score, often exceeding 0.9; meanwhile, the confidence scores in identifying several nuts fell below the 0.5 threshold. Based on YOLO’s results, the corresponding templates were generated using latent space template searching, and YOLO’s results were refined through those generated templates.

**Figure 23:**
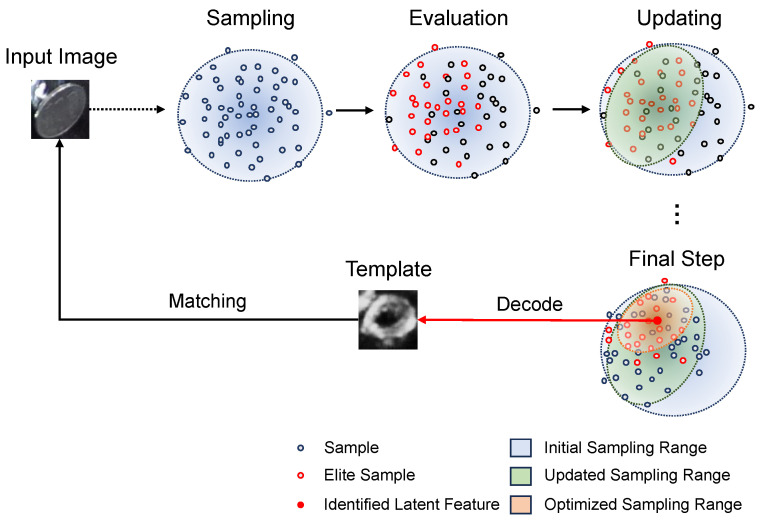
Template searching process in latent space by CEM. Each CEM optimization step consists of three processes: sampling, evaluation, and updating. After several CEM optimization steps, if no template is found that results in a template matching score exceeding the threshold the input image can be considered as not the target object.

Figure 23 demonstrates the process of refining YOLO’s results. If the matching score exceeds the threshold, the object can be considered as the target. Conversely, even after CEM optimization process, if no template is generated that exceeds the threshold, the input image can be considered as not containing the target object. Additionally, since YOLO provides the candidates, the CEM process does not need to scan the entire camera frame, reducing the time cost to less than 0.35 seconds for one template generation.

**Figure 24:**
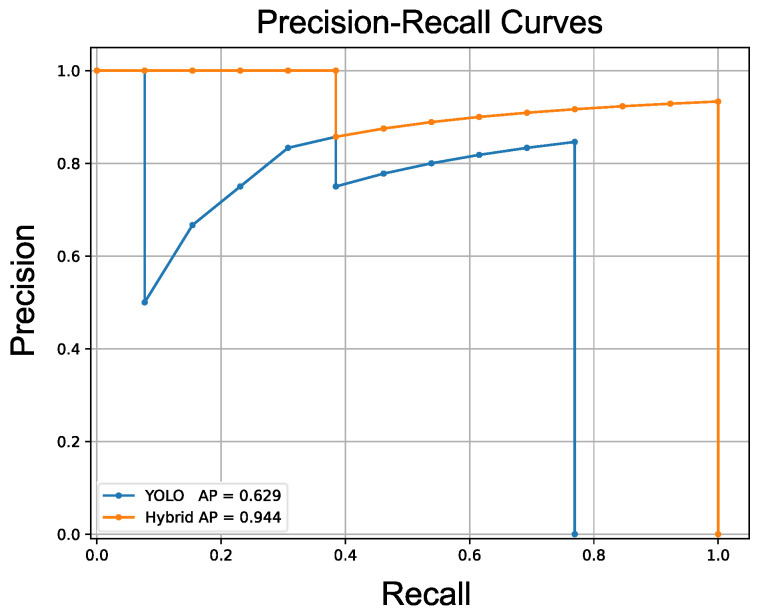
Precision–recall curves of two methods. The precision–recall curves of the two methods show that YOLO achieved an AP of 0.629, while our hybrid method achieved an AP of 0.944. The hybrid method achieved higher precision and recall than YOLO, demonstrating its better performance compared to YOLO.

To evaluate the detection performance of YOLO and the hybrid method, average precision (AP) was used as the evaluation metric. AP measures two aspects of the detection model: precision and recall. Precision reflects the model’s ability to distinguish between nuts and coins, while recall represents the model’s ability to detect nuts even in varying states. Ambiguous detection results with scores below the threshold are not counted as detected results. Figure 24 demonstrates the example’s precision–recall curves of both methods, highlighting the improvement achieved through latent space template searching.

Ten trials were conducted for the evaluation, and Table 8 illustrates the results. In each experiment, the nuts were arranged randomly, and two coins were randomly placed on the nuts for each experiment.The hybrid detection system achieves a mean AP of 0.941 with a standard deviation of 0.081, while YOLO only attained a mean AP of 0.768 with a standard deviation of 0.113. This indicates that the integration of latent space template searching effectively improves YOLO’s performance in the experiment task.

#### 4.2.2. Validation with VAE Reconstruction Approach

**Figure 25:**
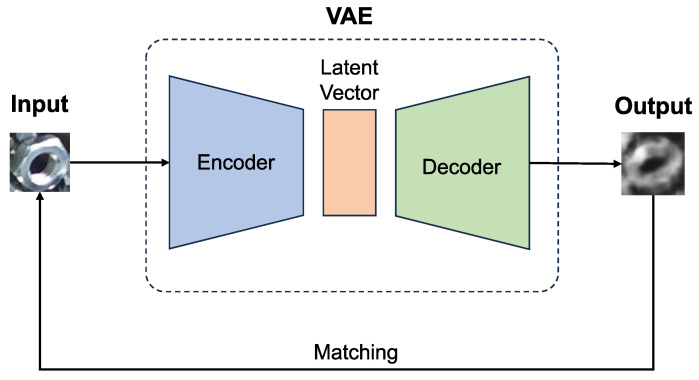
The VAE reconstruction approach for abnormal detection. An untrained input can be reconstructed to resemble the VAE’s training data. By matching the input and the output, it can be determined whether the input belongs to the training domain or not. However, an object with an untrained factor combination can also cause a lower matching score, which may affect detection performance. In the figure, the matching score is 0.410, which is below the threshold of 0.5.

The VAE is frequently used in abnormal detection, and Figure 25 shows the architecture. In this approach, YOLO’s detection results are reconstructed by the VAE and then matched with both the input and reconstructed images. An untrained input can be reconstructed to resemble the VAE’s training data. By matching the input and the reconstructed image, it can be determined whether the input belongs to the training domain or not [31].

**Figure 26:**
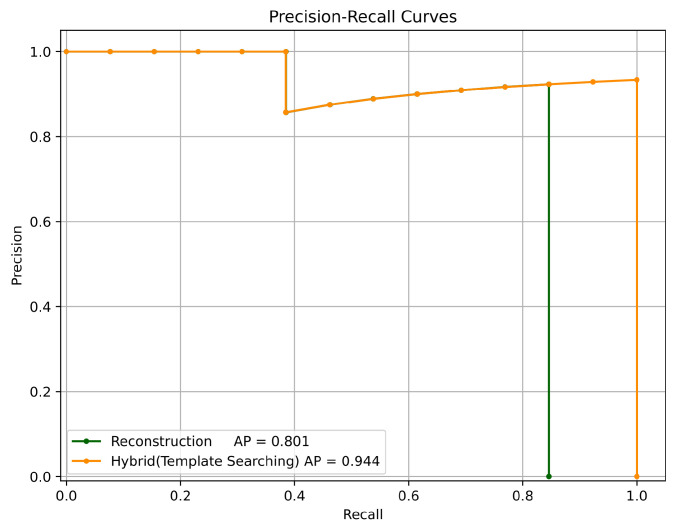
Precision–recall curves of two template generation approaches. The reconstruction approach achieved an AP of 0.801, while the proposed searching method reached an AP of 0.944.

To compare the VAE reconstruction approach with our proposed latent space searching method, the same test camera frame as described in Section 4.2.1 were used. Figure 26 shows the results for the two approaches, and the results indicate that while the reconstruction approach demonstrates an ability for abnormal detection, it struggles to improve recall.

Ten trials with same experiment data and with 100-yen coins were conducted for the evaluation, and Table 9 illustrates the results.

**Figure 27:**
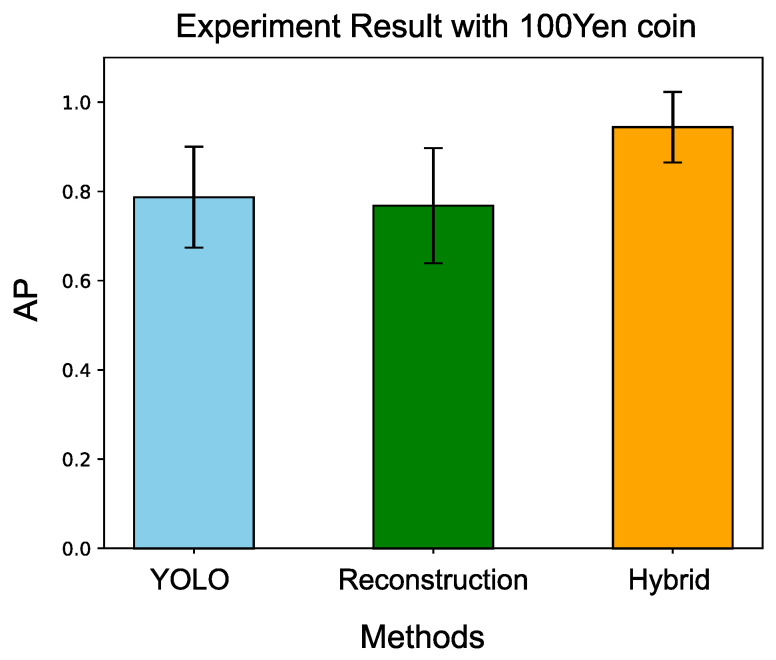
Comparison with three different methods. Our proposed hybrid method demonstrates the best performance among the compared methods.

Figure 27 compares the different methods: YOLO, the reconstruction approach, and the hybrid (template searching) approach. The VAE reconstruction approach does not improve YOLO’s AP efficiently, while our proposed method demonstrates a significant improvement over YOLO.

#### 4.2.3. Validation with Washer

**Figure 28:**
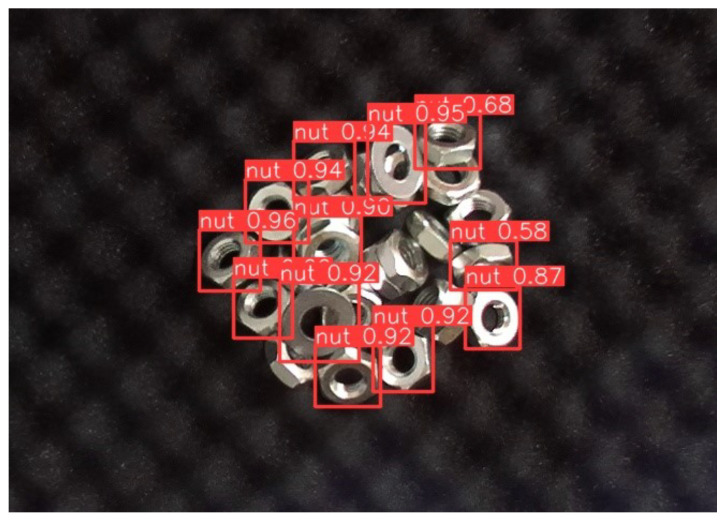
YOLO’s detection result with the washer. The washers achieved confidence scores exceeding 0.90, which were even higher than those for the nuts.

**Figure 29:**
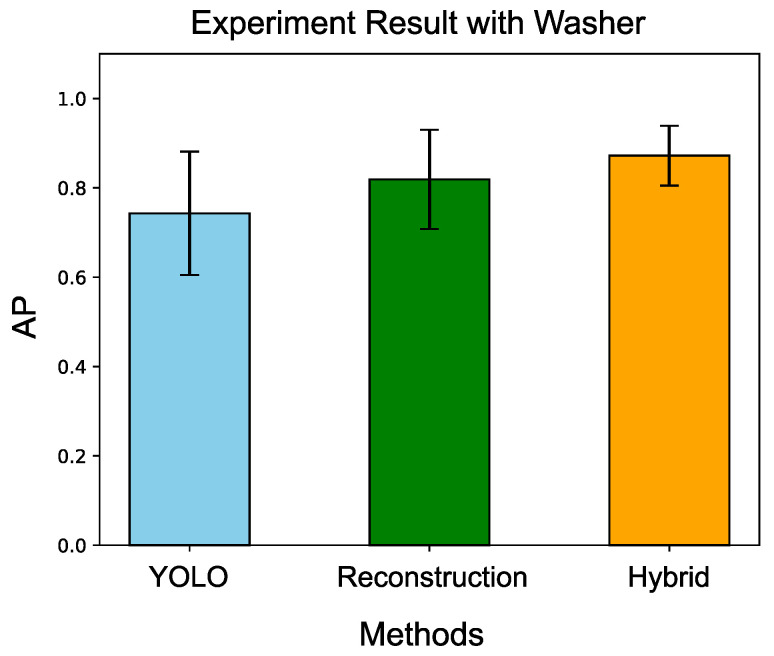
Comparison with three different methods. Our proposed hybrid method demonstrates the best performance among the compared methods.

To further validate the approach, a washer, which has a more similar appearance to a nut, was used. Figure 28 shows the YOLO’s detection result with washers. The experiment settings were the same as the experiment with the coins. Ten trials were conducted, and Table 10 presents the experimental results. Figure 29 summarizes the comparison results among the three methods. Although the washer is more challenging to distinguish compared to the coin, the proposed method still demonstrated the best performance among the compared approaches.

## 5. Discussion

### 5.1. Additional Learning for Matching Performance Improvement

**Figure 30:**
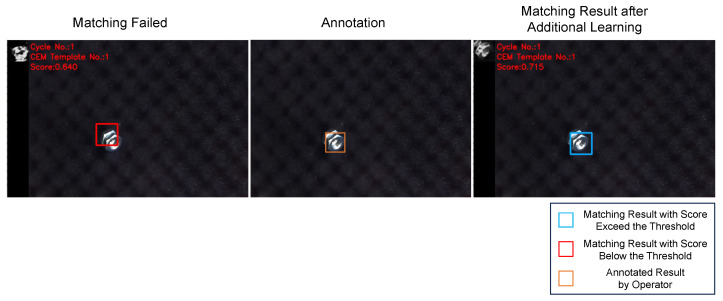
Matching performance improvement after additional learning.

In the experiment, unexpected template generation led to inaccurate matching. To analyze the reason behind this issue, we recreated the situation as shown in Figure 17. By annotating the incorrect matching and incorporating this annotation into the training data, the proposed method generated a template more closely resembling the target object, thereby improving the matching performance as illustrated in Figure 30.

**Figure 31:**
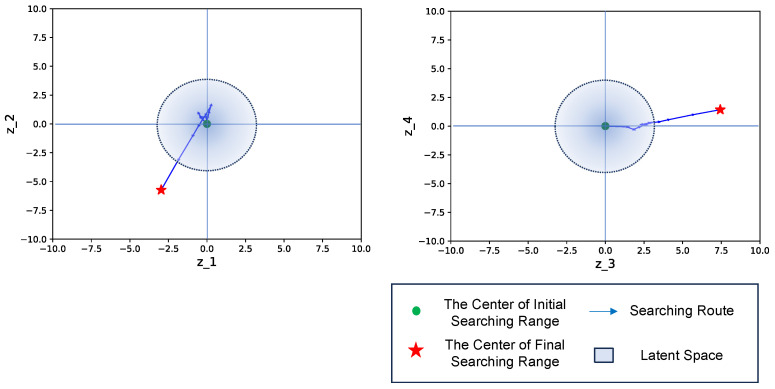
The searching process before additional learning. An untrained situation resulted in the expected latent feature outside the trained latent space, leading to the generation of an unexpected template.

**Figure 32:**
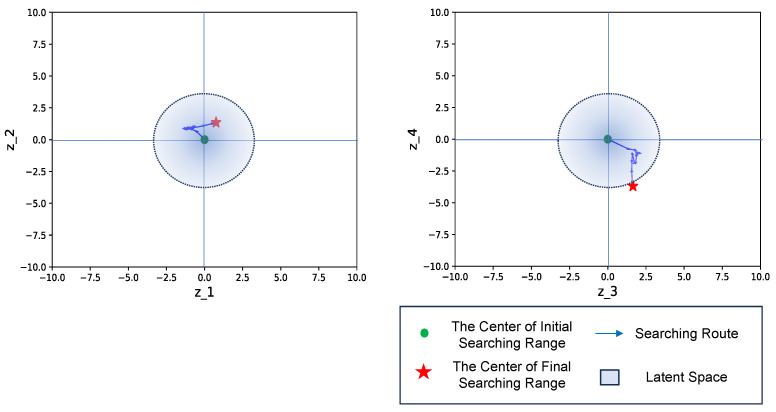
The searching process after additional learning. After incorporating the annotated result, the search trajectory of the latent feature shifted closer to the retrained latent space. Consequently, the generated template exhibited improved matching performance.

Before the additional learning, the untrained scenario led to the latent feature being situated outside the trained latent space, resulting in the generation of an unexpected template. The VAE learned the latent space by sampling from a standard Gaussian distribution, which suggests that the number of latent vectors typically falls within the range of around 3.0, according to the empirical rule. However, in an untrained situation, the expected latent feature may deviate from the trained latent space, leading to unexpected outcomes, as shown in Figure 31. After incorporating the annotation results into the training data and retraining the VAE model, we observed that the trajectory of the latent feature remained within the retrained latent space, resulting in the generation of a template with improved matching performance, as illustrated in Figure 32.

### 5.2. The Limitation of the NCC Similarity Measure

In this study, NCC was employed as the similarity measure for template matching. Since NCC focuses solely on the correlation between the template and the candidate window, it becomes challenging to accurately discern the object’s spatial relationship when occlusion occurs. For instance, the failure case shown in Figure 17 indicates that even though the template achieved a high matching score, the result was not suitable for grasping.

The integration with YOLO addressed the spatial challenges of NCC. YOLO was trained by labeling nuts that can be grasped by the robot, enabling it to detect only graspable nuts. This allows NCC to concentrate on measuring the difference between YOLO’s results and the generated template.

### 5.3. Matching within the Latent Space

**Figure 33:**
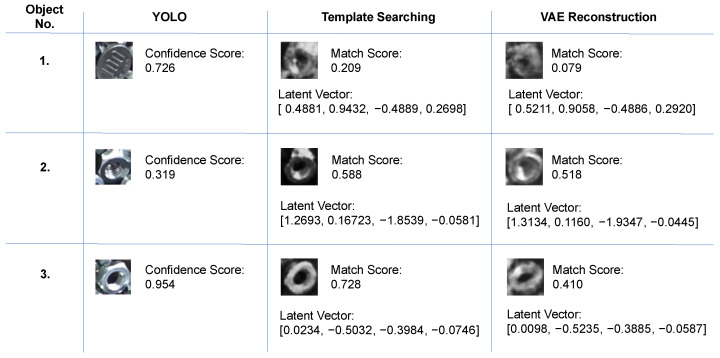
Templates and their latent vectors generated by various approach. Based on YOLO’s results, corresponding templates were generated by template searching and the VAE reconstruction approaches. The results indicate that both the input factors and their combinations are critical.

Since the latent space represents the variations in the training data, it might seem straightforward to match within the latent space by measuring the deviation of the latent vector from the prior. However, there are two main issues with this approach and Figure 33 illustrates three typical examples.

Firstly, the factors learned by the latent space are unknown. Since the VAE’s encoder, which uses CNNs, compresses the data, it is difficult to identify the specific factors that the latent space has learned. For example, Object No.1, a coin with a confidence score of 0.726, has a latent vector close to the prior, yet it is not a target object. Conversely, Object No.2, a nut with a confidence score of 0.319, has a latent vector that deviates from the prior but belongs to the target object. Therefore, relying solely on matching within the latent space may impact detection performance, making it essential to consider the combination of factors as well.

Secondly, due to the limited size of the customized training data, it is challenging for the latent space to learn all possible factor combinations. For example, in the case of Object No.3, the VAE reconstructed the input image with a different state, resulting in a lower matching score. In contrast, our proposed template searching method thoroughly explores all possible factor combinations in the latent space, finding an optimized latent vector similar to the input image’s factor combination and achieving a high matching score by its generated template.

Matching solely within the latent space only determines whether the input image is within the training domain, without accounting for factor combinations. Given the limited size of the customized training data, covering all possible factors and their combinations is challenging. While finding the exact factor combination of the input may be difficult, our proposed method can identify factor combinations close to the input. This approach allows it to find an optimal factor combination and generate a template that closely reflects the ground truth, thereby improving the reliability of the detection result.

## 6. Conclusions and Future Work

In this study, we proposed a novel template searching method that uses the VAE to induce the templates’ features into a low-dimensional latent space and integrates the CEM to generate an adaptive template dynamically within this latent space. The experimental results show that our proposed method can achieve a success ratio over 90% in bin-picking applications and successfully clear all tasks under various experimental conditions, demonstrating its effectiveness and robustness compared to conventional linear template searching strategies.

Furthermore, we integrated our proposed template searching method with YOLO, which enhanced its detection performance. The initial detection with YOLO addresses the limitations of template matching related to spatial issues and improves the efficiency of the searching process. Meanwhile, the latent space template searching approach identifies optimal factor combinations and generates a template that closely reflects the ground truth, thereby improving the reliability of the detection result.

For future work, we plan to test a wider variety of objects commonly used in manufacturing and to integrate our method into the manufacturing line to validate its performance in real-world applications.

## Figures and Tables

**Table 1:** Template update time consumption for each strategy.

Strategy	Time (second)
150 templates	1.033±0.001
300 templates	2.077±0.009
600 templates	4.183±0.009
1500 templates	10.512±0.042
Ours	2.089±0.559

**Table 2:** Experiment results for different object’s poses using our proposed method.

Trial	Success (%)	% Cleared	PPH
1	100	100	220
2	100	100	223
3	100	100	221
4	100	100	224
5	100	100	224

**Table 3:** Experiment results by different strategies.

Strategy	Success (%)	% Cleared	PPH
150 Templates	100	99.2	238
300 Templates	100	100	239
600 Templates	100	100	219
1500 Templates	100	100	209
Ours	100	100	222

**Table 4:** Experiment results for varying background appearances by proposed method.

Trial	Success (%)	% Cleared	PPH
1	92.8	100	206
2	90.9	100	204
3	92.6	100	207
4	90.3	100	211
5	90.0	100	208

**Table 5:** Comparison with different strategies in varying background appearances condition.

Strategy	Success (%)	% Cleared	PPH
150 Templates	92.7	44.8	203
300 Templates	88.9	45.0	194
600 Templates	89.5	98.0	180
1500 Templates	92.8	100	147
Ours	91.3	100	207

**Table 6:** Experiment results for additional lighting source.

Trial	Success (%)	% Cleared	PPH
1	92.6	100	213
2	92.6	100	211
3	89.6	100	199
4	90.3	100	206
5	93.3	100	228

**Table 7:** Experiment results by different strategies in additional lighting source condition.

Strategy	Success (%)	% Cleared	PPH
150 Templates	80.1	32.8	171
300 Templates	80.4	37.0	177
600 Templates	84.4	80.0	172
1500 Templates	90.9	100.0	142
Ours	91.7	100.0	211

**Table 8:** Experiment results with 100-yen Coin.

Trial	${\mathrm{AP}}_{\mathrm{YOLO}}$	${\mathrm{AP}}_{\mathrm{Hybrid}}$
1	0.890	1.000
2	0.748	0.917
3	0.919	1.000
4	0.807	0.743
5	0.878	0.878
6	0.762	0.944
7	0.629	0.944
8	0.620	1.000
9	0.701	1.000
10	0.911	0.983

**Table 9:** Experiment results with different template generation approaches.

Trial	${\mathrm{AP}}_{\mathrm{Reconstruction}}$	${\mathrm{AP}}_{\mathrm{Hybrid}}$
1	0.900	1.000
2	0.497	0.917
3	0.827	1.000
4	0.666	0.743
5	0.680	0.878
6	0.747	0.944
7	0.801	0.944
8	0.889	1.000
9	0.762	1.000
10	0.911	0.983

**Table 10:** Experiment results with washer by three different approaches.

Trial	${\mathrm{AP}}_{\mathrm{YOLO}}$	${\mathrm{AP}}_{\mathrm{Reconstruction}}$	${\mathrm{AP}}_{\mathrm{Hybrid}}$
1	0.854	0.771	0.854
2	0.568	0.766	0.766
3	0.604	0.861	0.849
4	0.662	0.831	0.831
5	0.869	0.935	0.935
6	0.786	0.935	0.935
7	0.895	0.589	0.795
8	0.536	0.889	0.918
9	0.778	0.900	0.860
10	0.877	0.711	0.977

## Data Availability

The raw data supporting the conclusions of this article will be made available by the authors on request.
